# P294: Assessment of the quality of gastroscopes disinfection in digestive endoscopy center in Abidjan

**DOI:** 10.1186/2047-2994-2-S1-P294

**Published:** 2013-06-20

**Authors:** C Assi, MF Adeoti

**Affiliations:** 1 CHU od Cocody, Côte d'Ivoire; 2 University Felix Houphouet Boigny, Abidjan, Côte d'Ivoire

## Objectives

To evaluate the practices of cleaning and disinfecting gastroscopes in digestive endoscopy centers in Abidjan.

## Methods

This was a prospective, multicenter, cross-sectional six-month study. All centers agreeing to participate in the study and performing at least one gastroscopy / session / week were included. Questionnaires, observation of disinfection and bacteriological tests were performed in each center (decontamination tanks, gastroscope, storage location).

## Results

Of 30 endoscopy centers in the city of Abidjan, 11 (36.6%; 6 private and 5 public) were included. All procedures for decontamination and disinfection have been carried out manually. Gastroscope disinfection was carried out by unskilled personnel in endoscopy. Decontamination was performed with quaternary ammonium proteolytic enzyme and surfactant in 8 centers. 2% glutaraldehyde was used in 9 centers for disinfection. Nine centers used 4 trays. All centers were utilized external cleaning and swabbing of gastroscopes. The minimum time of 15 minutes for decontamination and disinfection was observed respectively in 7 and 9 centers. Rinsing (less than 5 minutes) was performed after decontamination in 10 centers and after disinfection in 11 centers. The rinse water was changed late in the session in which 10 centers in a single center after the passage of each patient. Injection of distilled water into the working channel was performed in two centers after the final rinse. The concentration recommended by the manufacturers of the products decontamination and disinfection was observed in 10 of the 11 centers visited. Samples were positive in 10 of 11 centers. Bacillus (16%) was the most commonly encountered germ. The two rinsing tanks were the most contaminated environments early (7 centers) and end of the session (7 centers).

## Conclusion

The technique of manual disinfection gastroscopes was poor in digestive endoscopy centers in Abidjan. Improvement requires staff training in gastroscope disinfection procedures.

## Competing interests

None declared

